# Myonecrosis secondary to *Clostridium Septicum *in a patient with Occult Colon Malignancy: a case report

**DOI:** 10.1186/1757-1626-1-300

**Published:** 2008-11-07

**Authors:** Michael A Gibson, Dimitrios V Avgerinos, Omar H Llaguna, Nitin D Sheth

**Affiliations:** 1 Department of Surgery, Beth Israel Medical Center, Albert Einstein College of Medicine, First Avenue at 16th Street, New York, New York, USA

## Abstract

**Background:**

Gas gangrene is a relatively rare event that is typically associated with history of trauma. A non-traumatic history of gas gangrene has been associated with *Clostridium septicum *and cecal malignancy.

**Case presentation:**

We present a case of a 54-year-old male patient who presented with myonecrosis secondary to *Clostridium septicum *septicemia and an occult cecal carcinoma.

**Conclusion:**

*C. septicum *and its association with malignancy should be considered in any patient suffering from myonecrosis without a history of trauma.

## Introduction

*Clostridium septicum *is an uncommon cause of myonecrosis, most prominent in immunosuppressed patients [1]. Few reports in the world literature have established its association with cancer of the gastrointestinal tract, as well as its high morbidity and mortality rates. We present a rare case of a male patient who presented with myonecrosis of the arm, with a cecal malignancy discovered during the subsequent hospitalization.

## Case presentation

A 54-year-old man with type II diabetes mellitus presented to the emergency department of our hospital with a two-day history of diffuse abdominal pain worse in the right lower quadrant, and mild pain over the left upper arm beginning a day prior to his presentation. The abdominal pain was associated with nausea, vomiting, anorexia, and multiple episodes of diarrhea. The pain was constant since its onset and had progressively worsened since the previous day prompting the patient's coming to the hospital. The patient also complained of having a fever over the past day. His past medical history included uncontrolled type II diabetes mellitus that was diagnosed 5 years prior. The patient had not seen a doctor since his diagnosis of diabetes. The patient showed little concern for his arm and denied any history of trauma or injection to the area.

On examination, he was in mild distress with a temperature of 39.8°C, pulse of 106/min, and a blood pressure of 119/62 mmHg. The abdomen was distended, with tenderness on the right lower quadrant. His left shoulder had an area of erythema and induration with crepitus that began to extend towards the antecubital fossa. Lab results were within normal limits, apart from elevated white blood cell count to 13800/mm^3 ^and serum glucose of 380 mg/dL.

**Figure 1:**
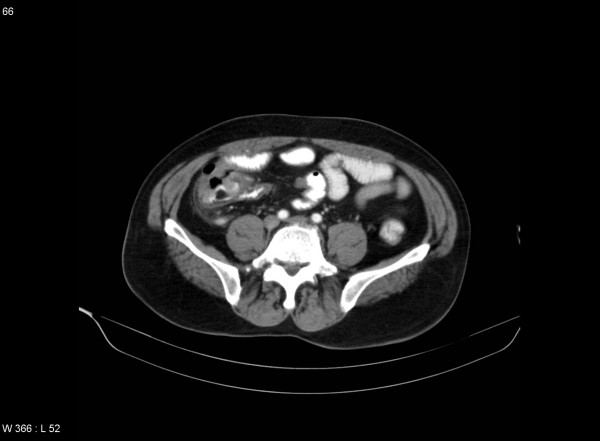
CT scan of the abdomen showing a polypoid mass of the cecum that almost completely obstructs the bowel lumen.

Abdominal x-ray films in the supine and erect position showed dilatation of small bowel loops within the lower mid abdomen with multiple air-fluid levels. A computed tomography (CT) of the abdomen revealed a nearly-obstructing polypoid mass of the cecum (see Figure 1), and CT of the left shoulder showed extensive emphysema in the soft tissue of the shoulder conforming to the shape of the deltoid muscle and cortical disruption along posterior aspect of the humeral head, leading to the diagnosis of necrotizing fasciitis.

Intravenous antibiotics including piperacillin/tazobactam, clindamycin, and vancomycin were administered. The patient was taken immediately to the operating room for extensive debridement of his left deltoid. All necrotic fascia and muscle were debrided and the patient was admitted to the surgical intensive care unit. Subsequent blood cultures and cultures taken from the soft tissue debrided during surgery revealed *Clostridium septicum*. During hospitalization there was a drop in the hemoglobin levels to 7.1 g/dL. A rectal exam was performed and was found to be guaiac positive. A subsequent colonoscopy revealed a cecal polypoid mass occupying more than 75% of the lumen. Serum tumor markers revealed a normal carcinoembryonic antigen (CEA) of 2.4 ng/mL. A right hemicolectomy was performed on the patient. Four out of seven lymph nodes were found to have positive metastasis of adenocarcinoma with colonic origin.

## Discussion

*Clostridium septicum *is believed to be a commensal organism of the intestine, however, in the setting of immunosuppression such as neutropenia, malignancy, typically colonic malignancy or acute leukemia, and poorly controlled diabetes, can become virulent. When *C. septicum *infection arises, it is typically bacteremic following spontaneous invasion from the gastrointestinal tract causing focal or disseminated infection. *C. septicum *is aerotolerant, unlike *C. perfringens *which requires an anaerobic environment to produce its toxins, and can infect normal tissue without history of trauma [1,2]. Seeding tends to involve the extremities and may infect more than one area [2].

*C. septicum *produces a rapidly spreading infection, with excruciating pain, edema, crepitance, bullae, and widespread muscle necrosis, often leading to death [2-4]. Infection may occur in the presence of trauma, but commonly presents without trauma. Predisposing factors to *C. septicum *infection include colon carcinoma, diverticulitis, gastrointestinal surgery, leukemia, lymphoproliferative disorders, chemotherapy, radiation therapy, neutropenia, and AIDS [2,5].

The association of *C. septicum *with malignancies of the colon, in particular of the cecum, has been well documented. The presence of a mucosal barrier disruption such as from malignancy allows invasion of *C. septicum *into the blood stream. When the malignancy outgrows its blood supply, an anaerobic environment is created which is ideal for *C. septicum *to proliferate [1,2].

In a study by Bretzke *et al *of 241 Clostridial infections, 7.8% (n = 19) were *Clostridium septicum *species. Overall mortality rates for Clostridial infections were 25%, while in Clostridium septicum species alone, mortality was 80%. Mortality in all *C. septicum *cases was associated with sepsis [6]. Larson *et al*, however, found *C. septicum *to be the responsible species in as high as 11.4% (n = 32) of all *Clostridial *infections (n = 281) with 56% mortality in comparison to 26% mortality in all Clostridial infections. *C. septicum *was also found to have an associated malignancy in 50% of cases, 75% of which were colonic, of these 40% were of the cecum. The remaining patients all had evidence of immunosuppression [7].

In diagnosis of *C. septicum*, blood cultures must be obtained, but aggressive resuscitation, surgical debridement, and intravenous antibiotic treatment are crucial to decrease mortality [8]. The majority of deaths occur within the first 24 hours [3]. Outcomes are dependent on early recognition and diagnosis which can be missed or delayed if Clostridial infection is not considered. *C. septicum *and its association with malignancy should be considered in any patient suffering from myonecrosis without a history of trauma.

Following debridement, involved areas must be exposed. Delayed primary closure or skin graft may be required after the infection is completely resolved. Patients surviving bacteremia and gangrene should have diagnostic studies to rule out pathology of the gastrointestinal tract. Metastatic spread may recur if the primary focus is not removed [4].

The presentation of the patient in this case was uncommon in that there was no complaint of excruciating pain at the site of myonecrosis. The patient had little concern for the mild pain in his upper arm displaying more distress secondary to his abdominal pain. The infection in this patient most likely began only hours prior to its discovery even though his abdominal symptoms had continued over the two previous days. The abdominal pain most likely signifies a disruption in the mucosal barrier of the patient's cecum, where the occult malignancy was found, allowing bacteremia and seeding to occur.

## Consent

Written informed consent was obtained from the patient for publication of this case report and accompanying images. A copy of the written consent is available for review by the Editor-in-Chief of this journal.

## Competing interests

The authors declare that they have no competing interests.

## Authors' contributions

DA and MG analyzed and interpreted the patient data. DA and OL performed the literature review, and was a major contributor in writing the manuscript. NS performed the final editing of the manuscript. All authors read and approved the final manuscript.
